# MicroRNA-375 inhibits laryngeal squamous cell carcinoma progression via targeting CST1

**DOI:** 10.1016/j.bjorl.2022.06.005

**Published:** 2022-07-06

**Authors:** Feng Dai, Zuojun Xie, Qiming Yang, Zhuanglong Zhong, Chun Zhong, Yongliang Qiu

**Affiliations:** Jiangxi Pingxiang People’s Hospital, Department of Otorhinolaryngology Head and Neck Surgery, Pingxiang, China

**Keywords:** miR-375, Laryngeal squamous cell carcinoma, Cystatin SN, Proliferation, Migration, Invasion

## Abstract

**Objective:**

This study aims to explore the effect and mechanism of miR-375 in Laryngeal Squamous Cell Carcinoma (LSCC) cell progression.

**Methods:**

LSCC cells (LSC-1 and TU177) were transfected with miR-375-mimic, miR-375-inhibitor or miR-375-mimic + oe-CST1. The expression of miR-375, CST1, MMP-2, and MMP-9 was measured. The effect of miR-375-mimic, miR-375-inhibitor or miR-375-mimic + oe-CST1 on cell biological functions, including cell proliferation, migration, invasion, and apoptosis, was also assessed. The potential relationship between CST1 and miR-375 was predicted by Jefferson software and validated by dual luciferase reporter gene assay.

**Results:**

Downregulated miR-375 expression was found in LSCC cells. Overexpression of miR-375 inhibited the viability and migration and promoted apoptosis of LSCC cells. Jefferson database and dual luciferase reporter gene assay confirmed that miR-375 directly targeted CST1. Overexpression of CST1 could reverse the anti-cancer effect of miR-375 overexpression in LSCC cells.

**Conclusion:**

Collected evidence showed that miR-375/CST1 axis was implicated in LSCC progression.

**Level of evidence:**

Level 3

## Highlights

• miR-375 is lowly expressed in LSCC cell lines.
• miR-375 regulates the proliferation, migration and invasion of LSCC cells.
• miR-375 targets CST1.
• miR-375 inhibits proliferation of LSCC cells by targeting CST1.

## Introduction

Laryngeal squamous cell carcinoma (LSCC) is a common type of Head and Neck Squamous Cell Carcinoma (HNSC).^1^ Currently, treatments including surgical resection, chemotherapy, radiotherapy, and immunotherapy are widely used in the cure of LSCC.^2^ Unfortunately, the overall survival rate has been decreased over the last couple of decades, as a large number of patients are diagnosed at an advanced stage and do not opt for surgical treatment.^3^ Therefore, understanding the mechanisms of LSCC progression and characterizing more reliable diagnostic and therapeutic biomarkers are critical for LSCC diagnosis and treatment.

MicroRNAs (miRNAs) are a cluster of single-strand noncoding RNAs that are endowed with the ability to regulate gene expression.^4^ There is ample evidence that miRNAs play a suppressing or promoting role in cancer growth by mediating the mRNAs encoded by anti-tumor genes or oncogenes.^5^ At present, scholars have identified some aberrantly expressed miRNAs in LSCC and elucidated their regulation on LSCC cell proliferation and aggressive phenotype.^6^, ^7^ Of these, miR-375-3p was reported to suppress the development of LSCC by targeting HNF1β.^8^ Previous studies showed that miR-375 suppressed LSCC development by regulating KLF4 expression,^9^ and inhibited the extracellular matrix degradation and invasive ability of HNSC cells.^10^ Moreover, clinical data have shown down-regulated miR-375 expression in laryngeal cancer tissues,^11^ and lower miR-375 expression was also associated with poor prognosis of HNSC patients.^12^ However, the underlying molecular mechanisms of miR-375-3p in LSCC are not well studied.

Cystatin SN (CST1) belongs to the type 2 cystatin superfamily and is involved in inflammation, cell cycle, cell senescence, tumorigenesis, and metastasis.^13^ It was reported that CST1 promoted pancreatic cancer growth.^14^ Also, CST1 was regulated by LET-7 in colorectal cancer, thereby affecting the proliferation ability of tumor cells.^15^ In analyzing Esophageal Squamous Cell Carcinoma (ESCC) samples, CST1 was upregulated in ESCC tissues^16^ and validated as a promising serological biomarker for the diagnosis of early ESCC,^17^ yet the role of CST1 in LSCC is elusive. Herein, Jefferson online software predicted a binding site of CST1 with miR-375. Therefore, we attempted to investigate whether miR-375 could regulate LSCC progression through a miRNA-mRNA network in relation to CST1.

## Methods

### Cell culture

Human LSCC cell lines (LSC-1, FaDu, FD-LSC-1, and TU177) and a Normal Laryngeal Epithelial Cell Line (NPEC) purchased from Procell Life Science & Technology Co., Ltd. (Wuhan, China) were cultured in RPMI-1640 medium (GIBCO, Thermo Fisher Scientific) added with 10% fetal bovine serum at 37 °C in an atmosphere of 5% CO_2_. When cell confluence reached 90%, cell passage was carried out, and the cell lines were screened by qRT-PCR.

### Cell transfection and grouping

miR-375 mimic (miR-375-mimic, 50 nM), miR-375 inhibitor (miR-375-inhibitor, 100 nM), CST1 overexpression plasmid (oe-CST1, 2 μg), and their corresponding negative controls (mimic-NC, inhibitor-NC, and oe-NC) were all purchased from GeneChem (Shanghai, China). Cell transfection was performed based on the instruction of the Lipofectamine 2000 kit (Invitrogen, Carlsbad, CA, USA). The following experiments were performed 48 h after the transfection.

### qRT-PCR

The extraction of Total RNA (Invitrogen, Car, USA) and the reverse transcription into cDNA were executed using Trizol and a PrimeScript RT kit (RR037A, Takara, Japan), respectively. Fluorescent quantitative PCR was performed per the instructions of SYBR®Premix ExTaqTM Ⅱ Kit (RR820A, TaKaRa). The cDNA templates were subjected to real time fluorescence quantitative PCR (ABI 7500, ABI, Foster City, CA, USA). The relative expression of target genes was internalized to that of GAPDH or U6 and calculated by the 2^−ΔΔCt^ method: ΔΔCt = ΔCt experimental group − ΔCt control group, ΔCt = Ct target gene − Ct internal control. Each experiment was run in triplicate. Relevant primers were designed by Shanghai Sangon Biotech (Table 1).

**Table 1:** qRT-PCR primer sequence.

Name of primer	Sequences (5ʹ–3ʹ)
miR-375-F	CGGGTTTGTTCGTTCGGCT
miR-375-R	GTGCAGGGTCCGAGGTATT
CST1-F	TGCGCCAAGAGACAGACAGAGAA
CST1-R	AGAGGGAGGCGATGCTACTGTTTA
GAPDH-F	AAATCCCATCACCATCTTCCAG
GAPDH-R	TGATGACCCTTTTGGCTCCC
U6-F	TGGTGTCGTGGAGTCG
U6-R	TGGTGTCGTGGAGTCG

F, Forward; R, Reverse.

### CCK-8 assay

The LSCC cells were inoculated onto 96-well plates with 100 μl diluted cell suspension (1 × 10^5^ cells/mL) per well. Each sample had triplicate wells. Then 10 μL of CCK-8 reagent (Tokyo, Dojindo, Japan) was appended into each well and incubated for 2 h after cells cultures for 0, 24, 48, and 72 h. The absorbance was measured at a wavelength of 450 nm.

### Transwell test

Cells (5 × 10^4^) were suspended in serum-free DMEM and inoculated onto transwell chambers coated with matrixgel (BD Biosciences, Bedford, MA, USA), and 600 μL medium containing 10% FBS was added into lower chambers. After 48-h incubation, the cells invaded to the lower surface of the membrane were fixed in 100% methanol and stained with 0.1% crystal violet. The non-invaded cells on the upper surface were removed with cotton swabs. Cells on the lower surface were photographed using a microscope (Olympus, Tokyo, Japan) in five random fields.

### Scratch test

The cells were inoculated onto 6-well plates at a density of 5 × 10^5^ per well. When grew to 90% confluence, the cells were scratched with a 200 μL pipette tip. The cells were washed three times with DPBS (14190250, Gibco, New York, USA), and the culture medium was refreshed with DMEM containing 2% FBS. The gap between the cells was observed under a low-power contrast microscope (Olympus MK, Tokyo, Japan). Cells in the same field were photographed after continuous culture for 24 h, and the change of the scratch width was observed. Migration rate = (0 h scratch distance − 24 h scratch distance)/0 h scratch distance. The experiment was run in triplicate.

### Flow cytometry

After transfection for 48 h, cells were digested with 0.25% trypsin (without EDTA) (YB15050057, Yubo Biotechnology Co., Ltd., Shanghai, China), and then collected for centrifugation with the supernatant discarded. After that, the cells were washed three times with cold PBS before centrifugation again and the supernatant was removed. Annexin-V-FITC apoptosis assay kit (K201-100, Biovision, USA) was used for measurement. The cells (1 × 10^6^) were suspended with 100 μL of PI solution, and mixed. After 15 min of incubation, the cells were mixed with 1 mL HEPES buffer (PB180325, Procell). Cell apoptosis was measured by flow cytometry. The experiment was repeated three times.

### Western blot

Enhanced RIPA cell lysis (Wuhan Boster Biological Technology., Ltd., Wuhan, China) containing protease inhibitors was used for cell lysis, and the protein concentration was determined using a BCA protein quantitative kit (Boster). The extracted protein was separated by 10% SDS-PAGE and transferred onto PVDF membranes before being blocked with 5% BSA for 2 h to block non-specific binding. Diluted primary antibodies of MMP-2 (ab92536, 1:1000, Abcam, Cambridge, UK), MMP-9 (ab76003, 1:1000, Abcam), mouse anti-human CST1 (ab68329, 1:1000, Abcam), and GAPDH (ab9485, 1:2500, Abcam) were added for incubation at 4 °C overnight. Then the protein was washed and incubated with HRP labeled goat anti-rabbit secondary antibody (ab6721, 1:2000, Abcam) or rabbit anti-mouse secondary antibody (ab6728, 1:2000, Abcam) for 1 h, followed by treatment with ECL solution (EMD Millipore company, USA) at room temperature for 1 min. The membrane was exposed to X-Ray films for 5–10 min for color development. Image J analysis software (National Institutes of Health, NIH) was used for gray quantification of western blot images, with GAPDH as the internal reference. Each group had three parallel experiments.

### Dual-luciferase reporter assay

Jefferson database (https://cm.jefferson.edu/) was implied to predict the binding site of miR-375 with CST1, according to which the wild-type fragments and mutant fragments were designed and inserted into pMIR-report plasmids. The Wild-Type luciferase Reporter Plasmid (WT-CST1) and mutant luciferase reporter plasmid (MUT-CST1) were co-transfected into HEK-293T cells with miR-375-mimic or mimic-NC (Shanghai North Connaught Biotechnology Co., Ltd., Shanghai, China). After transfection (48 h), the cells were collected and lysed before centrifugation for 3–5 min. The supernatant was collected to determine the luciferase activity using the luciferase assay kit (Dual-Luciferase Reporter Assay System, Promega, USA). The ratio of target luciferase activity and reference luciferase activity was used as the relative luciferase activity, and the luciferase activity was measured by a fluorescence detector (Promega). Three independent experiments were carried out.

### Statistical analysis

Data were analyzed by GraphPad prism 6 software and expressed as mean ± standard deviation (Σ±s). When two groups were analyzed, statistical comparisons were made by using *t*-test. One-way analysis of variance was adopted followed by Tukey’s multiple comparisons test for comparisons among more than two groups. *p* < 0.05 was thought to have statistical significance.

## Results

### miR-375 was lowly expressed in LSCC cells

**Figure 1:**
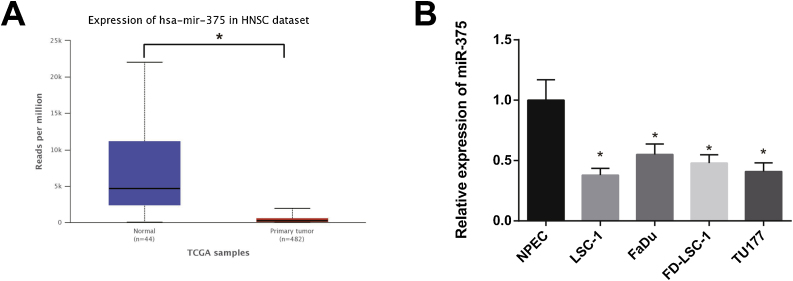
miR-375 expression was downregulated in LSCC cell lines. (A) lower expression of miR-375 in HNSC tissues was shown in TCGA database when compared with normal tissues. (B) qRT-PCR was used to measure the expression of miR-375 in human LSCC cell lines. * Compared with NPEC, *p* < 0.05. HNSC, Head and Neck Squamous Cell Carcinoma; LSCC, Laryngeal Squamous Cell Carcinoma.

TCGA database showed that miR-375 was decreasingly expressed in HNSC (Fig. 1A). As demonstrated in Fig. 1B, qRT-PCR results revealed that the expression of miR-375 in LSC-1, FaDu, FD-LSC-1, and TU177 cells was clearly lower than in NPEC cells, especially in LSC-1 and TU177 cells (p 0.05). Therefore, LSC-1 and TU177 cells were selected for subsequent experiments.

### miR-375 regulated the malignancy of LSCC cells

**Figure 2:**
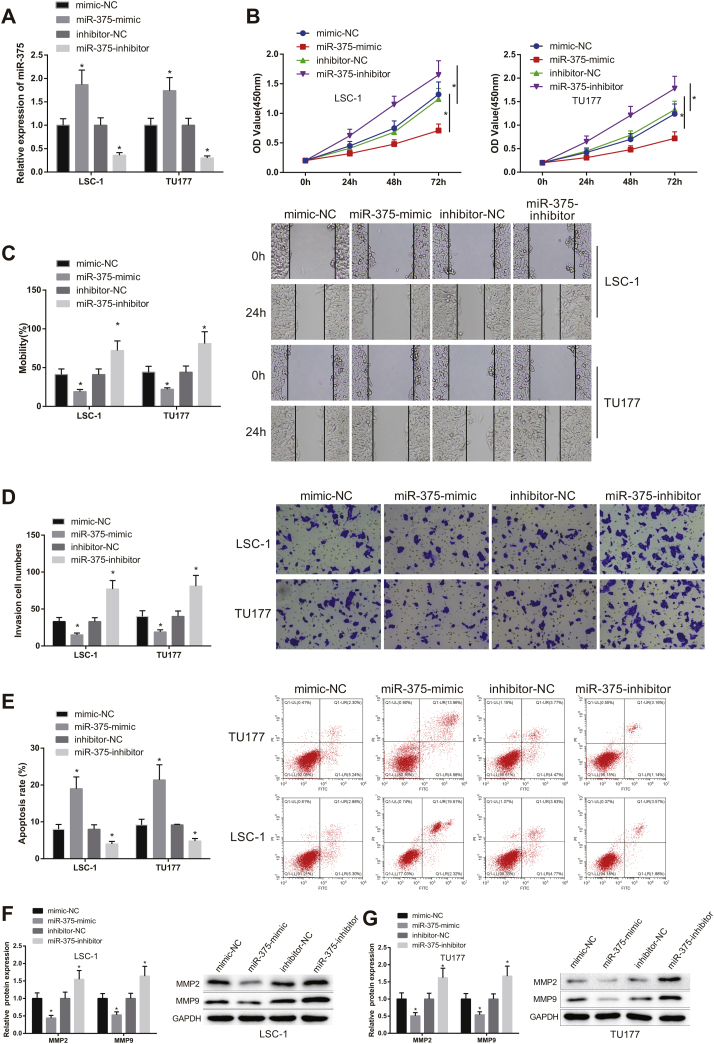
miR-375 overexpression in LSCC cells resulted in suppressed migration and elevated apoptosis. miR-375-mimic, miR-375-inhibitor or their controls were transfected into human LSCC cell lines LSC-1 and TU177. (A) the expression of miR-375 was tested by qRT-PCR. (B) cell viability in each group was measured by CCK-8 assay. (C—D) cell invasive and migration in each group were evaluated by scratch and transwell tests. (E) cell apoptosis in each group was measured by flow cytometry. (F—G) the protein expression of MMP2 and MMP9 in each group was assessed by western blot. * Compared with mimic-NC group or inhibitor-NC group, *p* < 0.05. LSCC, Laryngeal Squamous Cell Carcinoma.

miR-375-mimic or miR-375-inhibitor was transfected into LSC-1 and TU177 cells. The transfection efficiency was measured, and the results displayed that miR-375 expression was clearly increased in miR-375-mimic group (vs. mimic-NC group) and decreased in miR-375-inhibitor group (vs. inhibitor-NC group) (Fig. 2A, *p* < 0.05).

The viability of LSCC cells in each group was measured by CCK-8 assay. The results showed that cell viability was signally inhibited in miR-375-mimic group compared with mimic-NC group, but markedly enhanced in miR-375-inhibitor group compared with inhibitor -NC group (Fig. 2B, *p* < 0.05). Results from scratch and transwell tests revealed that the invasive and migration abilities of cells were clearly decreased in miR-375-mimic group compared with mimic-NC group, but clearly increased in miR-375-inhibitor group compared with inhibitor-NC group (Fig. 2C‒D, *p* < 0.05). Moreover, flow cytometry results displayed that the apoptosis rate in miR-375-mimic group was clearly increased compared with mimic-NC group, while decreased cell apoptosis was found in miR-375-inhibitor group compared with inhibitor-NC group (Fig. 2E, *p* < 0.05).

Extracellular matrix degradation-related proteins, MMP-2 and MMP-9 were highly expressed in laryngeal carcinoma, overexpression of which was related to tumor migration and invasion.^18^ The expression of MMP-2 and MMP-9 in lSC-1 and TU177 cells was further measured by western blot. The results showed that the protein expression of MMP-2 and MMP-9 was obviously dropped in miR-375-mimic group compared with mimic-NC group, but elevated in miR-375-inhibitor group compared with inhibitor-NC group (Fig. 2F, *p* < 0.05). Above results indicated that upregulated expression of miR-375 inhibited the malignancy of LSCC cells and promoted cell apoptosis, while downregulation of miR-375 expression resulted in reverse patterns.

### miR-375 directly targeted CST1

**Figure 3:**
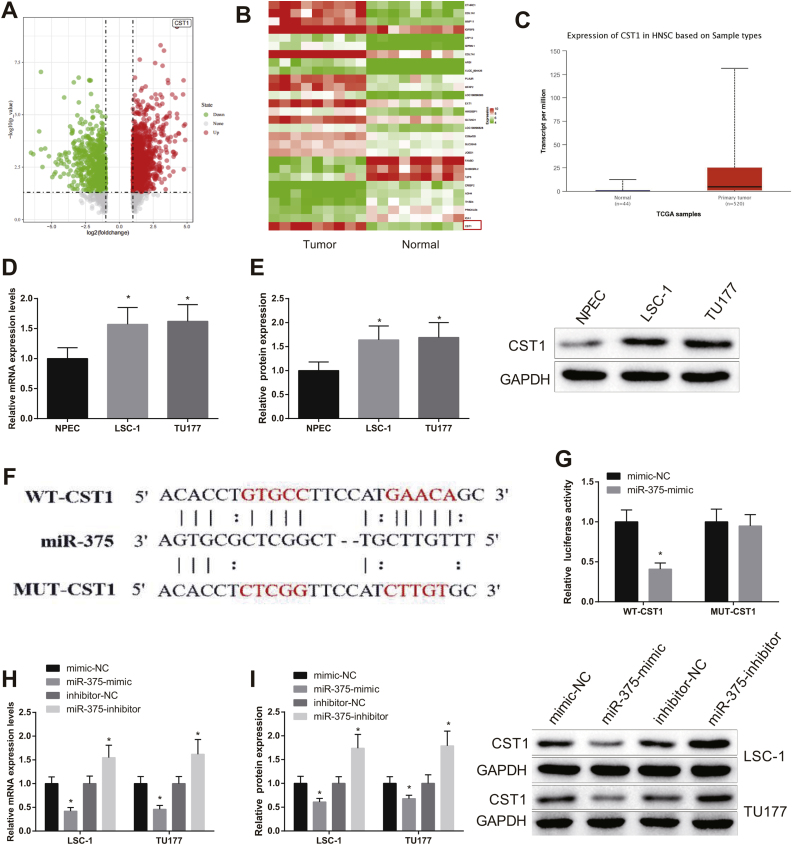
miR-375 targeted CST1. (A—C) GEO and TCGA database analysis showed that CST1 was highly expressed in HNSC. (D) the expression level of CST1 was examined by qRT-PCR and western blot. (E) Jefferson database showed that CST1 had direct binding sites with miR-375. G—H, qRT-PCR and western blot were used to detect the expression level of CST1. * Compared with NPEC or mimic-NC or inhibitor-NC group, *p* < 0.05. HNSC, Head and Neck Squamous Cell Carcinoma.

Differentially expressed genes in LSCC were analyzed by GEO database GSE84957 dataset and TCGA database, and it was found that CST1 was highly expressed in HNSC (Fig. 3A‒C). However, there was no study exploring the mechanism of CST1 regulation in LSCC. As reflected by qRT-PCR and western blot, the expression of CST1 in LSC-1 and TU177 cells was markedly higher than in NPEC cells (Fig. 3D‒E, *p* < 0.05).

Jefferson database showed that CST1 had a direct binding site with miR-375 (Fig. 3F). The binding relationship between miR-375 and CST1 was evaluated by dual luciferase reporter gene assay. The results showed that the luciferase activity of cells transfected with WT-CST1 was clearly decreased in miR-375-mimic group compared with mimic-NC group (Fig. 3G, *p* < 0.05). The luciferase activity of cells transfected with MUT-CST1 showed significant difference neither in miR-375-mimic group nor in mimic-NC group. qRT-PCR and western blot were used to assess the expression level of CST1 in cells after miR-375-mimic or miR-375-inhibitor was transfected. The experiment result indicated that the expression of CST1 in miR-375-mimic group was signally decreased compared with mimic-NC group, while that in miR-375-inhibitor group was dramatically increased compared with inhibitor-NC group (Fig. 3H‒I, *p* < 0.05). From these results, it was clear that miR-375 targeted CST1 in LSCC cells.

### miR-375 inhibited LSCC cell prliferation by targeting CST1

**Figure 4:**
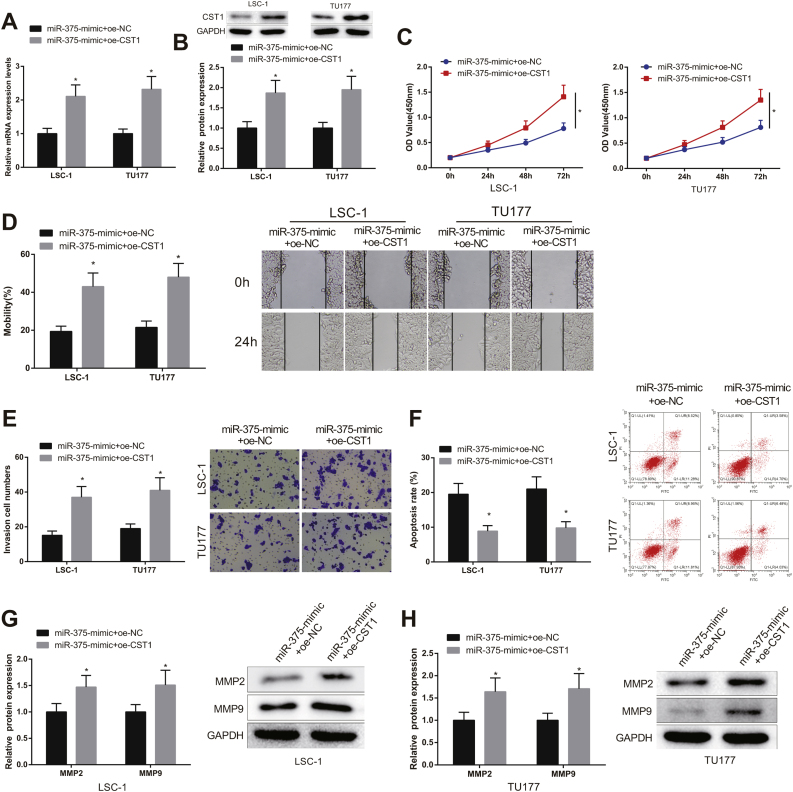
miR-375 suppressed CST1 to inhibit LSCC cell proliferation. (A—B) the expression level of CST1 was measured by qRT-PCR and western blot. (C) cell viability was tested by CCK-8 assay. (D—E) cell invasive and migration were measured by scratch test and transwell assay. (F) the apoptosis of cell in each group was evaluated by flow cytometry. (G—H) the protein expression of MMP2 and MMP9 in each group was assessed by western blot, * Compared with miR-375-mimic + oe-NC group, *p* < 0.05. LSCC, Laryngeal Squamous Cell Carcinoma.

LSC-1 and TU177 cells were introduced with miR-375-mimic + oe-CST1 and miR-375-mimic + oe-NC, and the impact of miR-375-mediated CST1 inhibition on the viability of LSCC cells was further explored. First, results from qRT-PCR and western blot showed that the expression of CST1 in miR-375-mimic + oe-CST1 group was memorably increased compared with miR-375-mimic + oe-NC group (Fig. 4A‒B, *p* < 0.05).

Moreover, CCK-8, scratch, and transwell tests revealed that the malignancy of LSCC cells in miR-375-mimic + oe-CST1 group was dramatically enhanced compared with miR-375-mimic + oe-NC group (Fig. 4C‒E, *p* < 0.05). To the contrary, LSCC cell apoptosis was significantly decreased in miR-375-mimic + oe-CST1 group compared with miR-375-mimic + oe-NC group (Fig. 4F, *p* < 0.05). Western blot results indicated that the expression of MMP-2 and MMP-9 in miR-375-mimic + oe-CST1 group was clearly increased compared with miR-375-mimic + oe-NC group (Fig. 4G‒H, *p* < 0.05). Taken together, miR-375 inhibited the growth and invasiveness and facilitated the apoptosis of LSCC cells by targeting CST1.

## Discussion

LSCC is an aggressive head and neck neoplasm derived from the epithelial tissue of laryngeal mucosa.^19^ Nowadays, exploring the underlying molecular mechanism and identifying the regulatory factors of LSCC have attracted the interest of many researchers.^20^ Several miRNAs have been considered markers for the early diagnosis and prognosis of LSCC patients, such as miR-196b and miR-17-5p.^21^, ^22^ In our study, miR-375 was found to be low expressed in LSCC cell lines, and miR-375 overexpression led to suppressed cell proliferation, migration and invasion, as well as decreased expression levels of MMP-2 and MMP-9. In addition, we identified miR-375 directly targeted CST1, and miR-375 inhibited LSCC cell progression by targeting CST1.

Initially, by analyzing TCGA database, we found miR-375 expressed modestly in HNSC. Jia-Yuan et al. showed that overexpression of miR-375 suppressed the development of nasopharyngeal carcinoma.^23^ In esophageal Cancer (EC), enhanced expression of miR-375 attenuated EC cell biological activities.^24^ Also, miR-375 had the inhibitory effect on cell proliferation in Oral Squamous Cell Carcinoma (OSCC).^25^ Additionally, a previous study has shown that the miR-375/SLC7A11 axis suppresses viability and invasive of OSCC cells.^26^ The present work validated that upregulation of miR-375 inhibited LSCC cell viability and repressed cell apoptosis. Consistent with our results, miR-375-3p was reported to bind HNF1β to inhibit cell cycle, glucose consumption and lactate production in LSCC cells.^8^

To explore the downstream target of miR-375, the potential targets of miR-375 were predicted by online software and CST1 was confirmed to be its target gene in LSCC cells. As a tumor-related factor, CST1 has been reported to be overexpressed in gastric cancer cells^27^ and breast cancer cells.^28^ Consistent with this, CST1 was obviously elevated in LSCC cells in the present study. Following experiments in this study further identified that miR-375 directly targeted CST1 to inhibit the aggressiveness of LSCC cells and promote cell apoptosis. The same working mechanism has also been reported in previous studies. For example, the anti-tumor role of miR-370 was also found in LSCC by targeting Forkhead Box M1.^29^ In addition, miR-4497 also reported to suppress LSCC progression by negatively modulating GBX2 expression.^30^

Through bioinformatics analysis, the differential expression genes in LSCC were relevant to the extracellular matrix and the related structures and pathways, indicative of the significant role of extracellular matrix in LSCC progression.^20^

Matrix Metalloproteinases (MMPs) are zinc-dependent proteolytic enzymes capable of decomposing basement membranes and extracellular matrix components, which is a prerequisite for tumor cell invasion.^31^ MMP-2 and MMP-9 are recognized as major contributors to the proteolytic degradation of extracellular matrix during tumor invasion.^32^ Elevated MMP-9 level has been shown to positively correlate with invasive and metastatic potentials in hepatocellular carcinoma cells and hypopharyngeal carcinoma.^33^, ^34^ In this study, overexpression of miR-375 was verified to reduce the expression of MMP-2 and MMP-9, while miR-375 inhibition led to significant increases in MMP-2 and MMP-9 protein levels, indicating miR-375 may also regulate extracellular matrix degradation in LSCC. Moreover, rescue experiments also showed that upregulation of CST1 could counteract the suppressive effect of miR-375 overexpression on MMP-2 and MMP-9. Taken together, it was speculated that miR-375 may regulate CST1 to mediate extracellular matrix degradation so as to suppress LSCC progression.

## Conclusions

In summary, miR-375 was down-regulated but CST1 was increased in LSCC tissues, and miR-375 inhibited the aggressive phenotype of LSCC cells and promoted cell apoptosis by targeting CST1. Additionally, the miR-375/CST1 axis may have a certain relationship with extracellular matrix degradation of LSCC cells, but more evidence is required to validate this speculation. Our findings provide a novel insight into the pathogenesis of LSCC, suggesting miR-375 may function as a potential therapeutic target for treatment of LSCC.

## Conflicts of interest

The authors declare no conflicts of interest.
